# Improving itaconic acid production through genetic engineering of an industrial *Aspergillus terreus* strain

**DOI:** 10.1186/s12934-014-0119-y

**Published:** 2014-08-11

**Authors:** Xuenian Huang, Xuefeng Lu, Yueming Li, Xia Li, Jian-Jun Li

**Affiliations:** Key Laboratory of Biofuels, Shandong Provincial Key Laboratory of Energy Genetics, Qingdao Institute of Bioenergy and Bioprocess Technology, Chinese Academy of Sciences, No. 189 Songling Road, Qingdao, 266101 China; Qingdao Kehai Biochemistry Co., Ltd, No. 198 Langyatai Road, Jiaonan, 266400 Qingdao, China; University of Chinese Academy of Sciences, Beijing, 100049 China

**Keywords:** Itaconic acid, *Aspergillus terreus*, Genetic engineering, Itaconate titer

## Abstract

**Background:**

Itaconic acid, which has been declared to be one of the most promising and flexible building blocks, is currently used as monomer or co-monomer in the polymer industry, and produced commercially by *Aspergillus terreus*. However, the production level of itaconic acid hasn’t been improved in the past 40 years, and mutagenesis is still the main strategy to improve itaconate productivity. The genetic engineering approach hasn’t been applied in industrial *A. terreus* strains to increase itaconic acid production.

**Results:**

In this study, the genes closely related to itaconic acid production, including *cadA*, *mfsA*, *mttA*, ATEG_09969, *gpdA*, ATEG_01954, *acoA*, mt*-pfk*A and *citA*, were identified and overexpressed in an industrial *A. terreus* strain respectively. Overexpression of the genes *cadA* (*cis*-aconitate decarboxylase) and *mfsA* (Major Facilitator Superfamily Transporter) enhanced the itaconate production level by 9.4% and 5.1% in shake flasks respectively. Overexpression of other genes showed varied effects on itaconate production. The titers of other organic acids were affected by the introduced genes to different extent.

**Conclusions:**

Itaconic acid production could be improved through genetic engineering of the industrially used *A. terreus* strain. We have identified some important genes such as *cadA* and *mfsA*, whose overexpression led to the increased itaconate productivity, and successfully developed a strategy to establish a highly efficient microbial cell factory for itaconate protuction. Our results will provide a guide for further enhancement of the itaconic acid production level through genetic engineering in future.

**Electronic supplementary material:**

The online version of this article (doi:10.1186/s12934-014-0119-y) contains supplementary material, which is available to authorized users.

## Background

Itaconic acid is on the DOE (Department of Energy) top 12 list of the biotechnologically produced key building blocks in chemicals production, and is a valuable monomer or co-monomer in polymers manufacturing [1,2]. In future, it can even replace methacylic acid which is presently produced by the petrochemical industry [3]. Since the 1960s it has been produced by fermentation with *Aspergillus terreus*. Although several other natural itaconate producers such as *Ustilago maydis* and *Pseudozyma antartica* have been discovered, they couldn’t compete with *A. terreus* based on the production level of itaconic acid [1].

**Figure 1:**
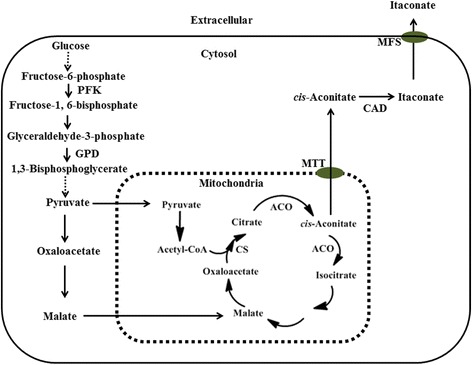
**Biosynthetic pathway of itaconic acid in** ***Aspergillus terreus*** **[** 1 **,** 5 **–** 7 **]** **.** The enzymes which were investigated in this study are shown in the figure. Abbreviations: PFK:6-phosphofructo-1-kinase; GPD: glyceraldehydes-3-phosphate dehydrogenase; ACO: aconitase; CS: citrate synthase; MTT: mitochondrial tricarboxylic transporter; CAD: *cis*-aconitate decarboxylase; MFS: major facilitator superfamily.

As far as the biosynthesis of itaconic acid is concerned, there has been a lot of debate for a long time, and it still hasn’t been fully established and understood [1,4–6]. It is generally accepted that the biosynthetic pathway occurs in two compartments: the cytosol and the mitochondria (Figure 1) [1,4,5,7]. The supposed pathway starts with glycolysis in the cytosol. Glucose is metabolized into pyruvate through the EMP pathway (Embden-Meyerhof-Parnas). Pyruvate is then either transported to the mitochondria and converted into acetyl-CoA, or carboxylated into oxaloacetate in the cytosol. Oxaloacetate is then transformed into malate, which is transported into the mitochondria by the malate/citrate antiporters. In the mitochondria, acetyl-CoA and oxaloacetate were condensed into citric acid by citrate synthase (CS). Citric acidis converted into *cis*-aconitate by aconitase (ACO), which is transported back into the cytosol with the help of mitochondrial tricarboxylic transporter(s) (MTT) and then decarboxylated into itaconate by *cis*-aconitate decarboxylase (CAD). Finally, itaconic acid is exported out of the cell likely via the di-carboxylic acid carrier.

Recently, Li *et al*. identified the gene cluster of the itaconic acid biosynthesis in *A. terreus* through a clone-based transcriptomics approach [5], comprising *mttA* (ATEG_09970) encoding the putative MTT which is suggested to function in transporting tricarboxylate such as citrate, *cis*-aconitate and isocitrate from the mitochondria to the cytosol and/or vice versa, *cadA* (ATEG_09971) encoding CAD which has been proved to be essential in the itaconic acid biosynthesis [8,9], *mfsA* (ATEG_09972) encoding the putative MFS (Major Facilitator Superfamily) transporter which is a diverse family of transporter proteins, transporting compounds ranging from sugars to organic acids, including dicarboxylic acids such as itaconic acid, and ATEG_09969 encoding the putative regulator protein which contains a zinc finger motif characteristic of the eukaryotic transcription factors. This is a great scientific breakthrough in the field of itaconic acid. However, the actual regulation of the itaconate biosynthesis has not yet been fully understood.

Despite 60 years of studies on itaconate production, the current titers are almost same as those of 40 years ago [10]. So far, mutagenesis and process optimization are still the main approaches to improve strains for higher itaconic acid production [1,11–13]. However, there is a need for further cutting down the cost of itaconate production, which could be realized by increasing the efficiency of itaconic acid production, achieving higher titers, and utilizing the cheap and sustainable substrates [1]. Genetic engineering can be applied to come to a breakthrough in this microbial production process*.*

There have been some examples of improving the production level of itaconic acid through genetic engineering. For instance, Tevž*et al.* reported that itaconic acid accumulation in *A. terreus* could be enhanced by deregulating the glycolytic flux that was achieved by introducing a gene encoding a highly active, citrate inhibition resistant 6-phosphofructo-1-kinase mutant (mt-PFK1) [14]; Expression of the *Vitreoscilla* hemoglobin gene in *A. terreus* and a fungal haemoglobin domain in itaconate-producing *A. niger* also improved the itaconic acid titer due to increased oxygen uptake [15,16]; Expression of *mttA*, *mfsA* and mt-*pfkA* (encoding the 6-phosphofructo-1-kinase mutant) in itaconate-producing *A. niger* increased itaconate productivity [16,17]; Expression of the itaconic acid cluster consisting of *cadA*, *mttA* and *mfsA* in *A. niger* led to a twenty-fold increase in itaconate yield compared to a strain expressing only *cadA* [18]. However, the *A. terreus* and *A. niger* strains used in the literature are non-industrial ones [14–19].

Moreover, some genes which are possibly related to itaconic acid production have been discovered. For example, the transcription levels of the *gpdA* gene encoding glyceraldehydes-3-phosphate dehydrogenase (GPD) (ATEG_09817) and ATEG_01954 (a predicted protein) dramatically increased 15.8-and 14.5-fold respectively under the optimal itaconate production conditions [5], and that of the *acoA* gene encoding aconitase (ACO) also increased under itaconate production conditions [7]. Furthermore, considering that citric acid is a key precursor of the itaconic acid biosynthesis, citrate synthase (CS), which is encoded by *citA* and catalyses an irreversible reaction, may play an important role in improving itaconic acid production.

The genetic engineering approach has been extensively used for filamentous fungi to improve the production level, and produce the novel tailored compounds or direct the synthesis of the desired compounds [20,21], but it has only been utilized in a few examples to improve itaconic acid production by non-industrial *A. terreus* and *A. niger* strains [14–19]. In the current study, the genes closely related to the itaconic acid biosynthesis were identified and used to improve itaconate production through genetic engineering of industrially used *A. terreus* strains.

## Results and discussion

### Gene cloning, construction of the expression cassettes, transformation and analysis

Since it has been realized that right compartmentalization of the overexpressed proteins is important for itaconate production [22], all candidate genes include their native signal peptides when they were cloned.

Mutant mt*-pfkA* from *A. niger *Co827 was made as reported [23]. The genes from *A. terreus* were amplified by PCR using the cNDA of *A. terreus *LYT10 as the template and the respective primer pairs. The coding sequences (CDSs) of most cloned genes showed high identities to the annotated ones in the genome sequence of *A. terreus* NIH 2624, and the sequence identities between them are >96% and >98% for CAD, MTT, CS and ATEG_01954 at the gene and protein levels respectively. However, the sequence identities between cloned *mfsA*, ATEG_09969, *gpdA*, *acoA* and the annotated ones are 80.5% to 93.2% and 81.5% to 92.8% at the gene and protein levels respectively. Since the functions of most annotated genes in the genome sequence of *A. terreus* NIH2624 were predicted only and have not been characterized yet, the big difference in CDSs between the cloned and annotated genes is possibly caused by designation of introns or/and exons or/and translation start sites or/and stop codons. The CDSs of *mfsA*, ATEG_09969, *gpdA* and *acoA* were deposited in GenBank with the accession numbers KF305087, KF305088, KC213825 and KF268034, respectively.

Most candidate genes were inserted into the vector pAN52-4 at the restriction sites of *Nco*I and *Bam*HI or *Hind*III respectively [24]. The obtained constructs were co-transformed with pAN7-1 harbouring the hygromycin resistance gene into *A. terreus* LYT10. The integrated target genes were confirmed by genomic PCR (Additional file 1: Figure S1). ATEG_01954 was cloned into the vector pG3H at the restrictions sites of *Pci*I and *Bgl*II [25], and the generated plasmid was linearized and transformed into *A. terreus* LYT10. The transformants were selected on PDA-SH plates. The copy numbers of the introduced genes were determined by Southern blot. Since it is still controversial about the relationship between the copy numbers and translation/transcription [26–28], the copy numbers were only determined for the chosen *cadA*- and *mfsA*-transformants (Additional file 2: Figure S2). Multi-copy genes were detected in most *cadA*- and *mfsA*-transformants, for example, 2-, 4-, 3-, 1-, and 2-copy of the *cadA* gene were integrated in the transformants *cadA*-2, −5, −18, −21 and −22 respectively (Additional file 2: Figure S2A), whereas 1-, 4-, 4-, 8-, and 2-copy of the *mfsA* gene were present in transformants *mfsA*-2, −10, −17, −12 and −24 respectively (Additional file 2: Figure S2B). It is worth pointing out that the endogenous *cadA* band was not observed in the *cadA*-2 transformant (Lane 2, Additional file 2: Figure S2A), indicating that homologous recombination occurred for it. Thus, one band should be from homologous recombination, and the other one is from random integration (Lane 2, Additional file 2: Figure S2A).Our results also indicated that there is no obvious relationship between the copy numbers of the introduced genes and the itaconate titer. Similar results were also observed by Tevž *et al.* when mt-*pfkA* was overexpressed in *A. terreus* [14].

The transformants which can grow healthily on PDA plates were tested for itaconic acid production by cultivation in shake flasks for 76 h at 37°C*. *Triplicate experiments were performed for each transformant, and were repeated for the better transformants at least once.

### Effects of respective overexpression of *cadA*, *mfsA*, *mttA* and ATEG_09969 in *A. terreus* on itaconic acid production

**Figure 2:**
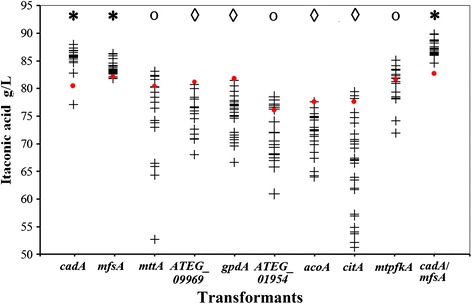
**Itaconic acid production by the transformants of different genes.** The transformants of the different genes were screened for itaconate production. Each transformant was grown in 50 ml IPM on a rotary shaker at 37°C for 76 hr in three individual flaks. The titers of itaconic acid produced were determined by HPLC. WT was represented by the solid red circle. *: statistically significant (ρ < 0.05, beneficial for itaconate production). ⋄: statistically significant (ρ < 0.05, adverse for itaconate production). ο: statistically non-significant (ρ > 0.05, no impact on itaconate production).

Remarkably, twenty-three out of twenty-four *cadA*-transformants produced higher itaconic acid titers than WT (Additional file 3: Figure S3A) (Figure 2), among which the best one – *cadA*-21 produced itaconic acid up to 88.1 g/L, 9.4% higher than the parental strain, indicating that overexpression of *cadA* in *A. terreus* is beneficial for itaconate production (T-test, ρ = 0.000) (Figure 2).These results demonstrated that introduction of the *cadA* gene contributed to itaconate production, and CAD was functional when overexpressed in *A. terreus* LYT10. Variations of the itaconate titers between the *cadA*-transformants were observed (Additional file 3: Figure S3A) (Figure 2), which might be possibly due to heterologous recombination in *A. terreus* and/or growth effects. In fact, this is the first time that *cadA* was overexpressed in *A. terreu*s, particularly an industrial *A. terreus* strain. Our results were consistent with the observation that the transcription level of *cadA* increased under the itaconic acid producing conditions and that of *cadA* was approximately five-fold higher in the high-producing strain than that in the wild-type strain [5,8], and also confirmed that CAD is a key enzyme involved in the itaconic acid biosynthesis and the CAD-catalyzed reaction might be a rate-limiting step for itaconate production. Because of its key role in the itaconic acid biosynthesis, *E. coli*, *Y. lipolytica* and *A. niger* have been engineered to produce itaconic acid by introducing the *cadA* gene [5,18,22,29–31]. However, the highest titer only reached 6 g/L [29], which is far below the current one of *A. terreus*.

All twenty-six *mfsA*-transformants showed slightly higher itaconic acid productivity than the parental strain (Additional file 3: Figure S3B) (Figure 2), in which the itaconate titer of the best one – *mfsA*-16 is 5.1% higher than that of WT, demonstrating that overexpression of *mfsA* in *A. terreus* is beneficial for itaconate production (T-test, ρ = 0.045) (Figure 2). The results were in good agreement with the reported ones where all 24 transformants produced higher itaconate titers than the parental strain while *mfsA* was introduced into itaconate-producing *A. niger*, and demonstrated that MFS might act as an itaconate exporter and play important roles in itaconic acid production [5,16]. Overexpression of *mfsA* could lead to faster transporting itaconic acid produced in the cytosol out of the cell, driving the metabolic flux toward itaconate biosynthesis, and enhancing the itaconate titer, though the improvement is not that high as overexpression of *cadA*.

The putative MTT (ATEG_09970), which might act as a citric acid and/or *cis*-aconitate carrier, was overexpressed in *A. terreus* LYT10. Most *mttA*-transformants showed slightly lower itaconic acid titers than WT (Figure 2), demonstrating that introduction of *mttA* into *A. terreus* is not beneficial for itaconate production (T-test, ρ = 0.24). Our results were consistent with the ones of Li *et al.*: only one transformant exhibited higher itaconic acid production level among eight *mttA*-transformants of itaconic acid producing *A. niger*, and they observed that the *mttA*-transformants showed the increased accumulation level of citric acid [16]. However, van der Straat *et al*. found that expression of *mttA* resulted in a twenty-fold increase in the secretion of itaconic acid in *A. niger* [18]. It is still not clear why different results were obtained for the *mttA*-transformants of *A. terreus* and *A. niger*. The fact that the equilibrium of the ACO-catalyzed reaction lies at the side of citrate with more than 90% and almost no citrate could be detected in cultures of itaconate producing *A. terreus* implies that very quick removal of *cis*-aconitate through MTT (or the *cis*-aconitate carriers) and/or CAD is very important for itaconic acid production [1]. A lot of studies on metabolite transport across the mitochondrial membrane have been carried out, for example, a novel transporter for dicarboxylates and tricarboxylates in the plant mitochondria has been identified [32], and a mitochondrial citrate transport protein from the yeast *S. cerevisiae* has been functionally characterized [33]. However, the mechanism of the mitochondrion carrier proteins related to organic acid transport in fungi is still not very clear. Accordingly, the function of MTT (ATEG_09970) awaits further research. Blumhoff *et al.* tried to address this issue in *A. niger* through co-overexpressing *cadA* and *acoA* in the cytosol and mitochondria [22].

When ATEG_09969 was introduced into *A. terreus* LYT10, most transformats produced slightly lower titers than WT (Figure 2), indicating that overexpression of ATEG_09969 into *A. terreus* is adverse for itaconate production (T-test, ρ = 0.014). Based on the speculation that ATEG_09969 might regulate itaconic acid production at the transcriptional level, overexpression of it could contribute to the higher itaconate production level [5].However, our results didn’t support this. Actually, ATEG_09969 is also directly linked to the lovastatin gene cluster and may play parts in the lovastatin biosynthesis, since it has been reported that lovastatin and its intermediates were not detected while ATEG_09969 (equivalent to *lovH* or *ORF13* in the lovastatin gene cluster) was rendered inactive via mutation [34]. In addition, Lai *et al.* found that the production levels of itaconic acid and lovastatin are inversely related [35]. Further study is required to clarify the function of ATEG_09969 in the itaconic acid biosynthesis.

### Effects of respective overexpression of *gpdA*, ATEG_01954, *acoA*, *citA* and mt-*pfkA* on the itaconic acid titers

When *gpdA*, ATEG_01954, *acoA* and *citA* were overexpressed in *A. terreus* LYT10 respectively, most transformants showed lower itaconate production level than the parental *A. terreus* strain (Figure 2). The effects of overexpression of *gpdA*, ATEG_01954 and *acoA* on the itaconate titers were not expected based on the transcription data [5,7], and the screening results were discussed as follows: (1) Li *et al*. also observed that a true *gpdA* transformant (containing 4 extra gene copies) showed lower itaconate production level than WT, but they didn’t explain why [19]. It has been realized that the requirement of an overall redox balance within the cell is a key factor determining the overall yields and productivities of a given product [36,37]. Since GPD is an NAD^+^ dependent enzyme, overexpression of GPD in *A. terreus* might result in the imbalance of the redox cofactors, which would possibly affect itaconate production. (2) ATEG_01954 is a predicted small protein of 189 amino acids, whose function is unknown, so further research is required to elucidate its performance, its function in particular. (3) Given the fact that overexpression of *acoA* might not only contribute to the *cis*-aconitate pool, but also possibly accelerated conversion of *cis*-aconitate into isocitrate, it might be understandable that introduction of extra copies of *acoA* showed no obvious impact on the itaconate titer. However, based on the results of the *cadA*-transformants, overexpression of *cadA* could drive carbon flux towards itaconic acid biosynthesis, and it is possible to improve itaconic acid production through co-expression of *acoA* and *cadA*. Blumhoff*et al.* found that co-overexpression of *acoA* and *cadA* in the mitochondria doubled itaconate productivity compared with strains overexpressing both enzymes in the cytosol [22]. In addition, it has been reported that overexpression of ACO in *Yarrowia lipolytica* did not result in a shift of the CA (citric acid) /ICA (isocitric acid) product pattern into the direction of ICA [38]. (4) The results of *citA*-transformants implied that the reaction catalysed by CS might not be rate-limiting or CS contributes little to the flux control in the itaconic acid biosynthesis. Likewise, overexpression CS in *A. niger* didn’t increase the citric acid production level [39].

It has been assumed that the strong anaplerotic reactions that replenish the pool of the TCA cycle intermediates would enhance the synthesis and excretion rate of itaconic acid, which could be realized by a highly active, citrate inhibition-resistant shorter PFK1 fragment (mt-*pfkA*) [14]. Only five out of nineteen mt-*pfkA-*overexpressing transformants produced slightly higher itaconic acid titers than WT (Figure 2). Statistically (T-test, ρ = 0.448), overexpression of mt-*pfkA* in *A. terreus* is not beneficial for itaconate production (Figure 2). So, the production level of itaconic acid caused by overexpression of mt-*pfkA* was not significantly enhanced as reported [14]. The different performance of the mt-*pfkA* transformants could possibly arise from the fact that the different hosts were used, a hyper itaconic acid producer (~80 g/L) was utilized in our case, whereas a poor one (13.5 g/L) was used by Tevž *et al.* [14].

### Effects of co-overexpression of *cadA* and *mfsA* in *A. terreus* on itaconic acid production

Inspired by the performance of the *cadA*- and *mfsA*-transformants, *cadA* and *mfsA* were co-expressed in *A. terreus* LYT10 to see if there are any additive beneficial effects on itaconic acid production. All 20 co-transformants of *cadA* and *mfsA* produced higher itaconate titers than the parental strain, among which the titer of the best one *cadA*/*mfsA*-19 is 8.7% higher than that of WT (Additional file 4: Figure S4) (Figure 2), demonstrating that co-expression of *cadA* and *mfsA* in *A. terreus* contributes to itaconate production (T-test, ρ = 0.000). Unexpectedly, the co-transformants didn’t exhibit the obvious additive beneficial effects. It appears that the co-transformants are slightly better than the *cadA*-transformants, and much better than the *mfsA*-transformants (Figure 2), further proving the importance of *cadA* initaconate production. These results might imply that the unknown bottlenecks or factors could limit the performance of the co-transformants and need to be further identified.

### Itaconic acid production by the transformant *cadA*-21 at the pilot scale

The best itaconic acid producer *cadA*-21 was tested at the demonstration scale (35 m^3^fermentor). A two-stage process including the vegetative and production phase was used [40]. The growth of the transformant *cadA*-21 was nearly not affected (data not shown). Itaconic acid produced by *cadA*-21 was analysed and quantified by HPLC (High Performance Liquid Chromatography). Based on the HPLC results, the titer of itaconic acid in the final fermentation culture of *cadA*-21 was similar to the parental strain, around 78.5 ± 2.2 g/L (Additional file 5: Figure S5) (Additional file 6: Figure S6A). However, it consumed glucose slightly more quickly (Additional file 6: Figure S6B). Thereby the production stage was shortened for around 3 hr, indicating that over-expression of *cadA* in *A. terreus* didn’t have negative effect on the metabolic flux towards itaconate biosynthesis, but slightly accelerated that process.

### The effects of gene overexpression on extracellular organic acids rather than itaconic acid

The concentrations of extracellular organic acids produced by some strains were determined (Table 1). The citrate concentrations were estimated to be below 0.01 mM, while pyruvate, lactate and oxalate could not be detected in all samples.

**Table 1:** **Extracellular organic acids (mM) produced by different strains** ^**a**^

**Strain**	**Itaconic acid**	***cis*** **-aconitic acid**	**Malic acid**	**α-ketoglutaric acid**	**Succinic aicd**	**Fumaric acid**
**WT**	621.9	0.058	1.8	9.1	10.2	0.081
***cadA*** **-21**	667.8	0.059	1.6	8.0	11.0	0.074
***mfsA*** **-2**	646.0	0.068	1.8	7.2	9.9	0.075
**mt** ***-pfkA*** **-5**	630.62	0.066	1.5	7.3	9.8	0.074
***mttA-16***	590.8	0.067	1.8	7.6	15.2	0.094
***citA*** **-7**	573.01	0.081	1.1	6.2	4.1	0.081
**ATEG_01954-16**	563.3	0.051	1.7	8.2	9.4	0.081
**ATEG_09969-14**	539.9	0.046	1.8	7.9	8.0	0.069
***acoA*** **-20**	535.85	0.055	1.2	6.6	7.9	0.070
***gpdA*** **-11**	527.3	0.014	1.7	6.6	7.5	0.080

^a^The citrate concentrations were estimated to be below 0.01 mM, while pyruvate, lactate and oxalate could not be detected in all samples.

Organic acids were separated on a Bio-Rad HPX-87H column by HPLC after 76-hr incubation, and detected by a refractive index detector and a UV–vis one (at 210 nm). The standard curves of all organic acids were established using 0.5 mM crotonic acid as an internal standard.

All selected strains showed lower α-ketoglutarate titers in comparison with the parental strain. The *cis-*aconitate titers were improved by overexpression of *citA*, *mfsA*, *mttA* and mt*-pfk*A, whereas it was greatly reduced by introduction of *gpdA*. The malate titers were significantly decreased by overexpression of *citA* and *acoA*. Overexpression of *mttA* led to significantly enhanced succinate titer, while most transformants showed lower succinate production level than WT, strain *citA*-7 in particular. The fumarate titers of most strains were not affected too much, except those of *mttA-*16, ATEG_09969-14 and *acoA*-20.

Our results demonstrated that accumulation of *cis-*aconitic acid is important for itaconate production and the titers of organic acids were affected by the introduced genes to different extent. The observation that the itaconate concentration (around 600 mM) is significantly higher than the citrate one (below 0.01 mM) reflected the special itaconate accumulation mechanism in *A. terreus*.

### The regulation of the itaconate biosynthesis in *A. terreus*

Based on the studies towards citric acid production by *A. niger*, Torres *et al.* used the biochemical systems theory coupled with constrained linear optimization to show that at least seven enzymes need to be co-expressed to achieve a significant increase in flux towards the citrate biosynthesis [41], and predicted that adjusting the step that removes the desired product constituents the most promising metabolic engineering strategy, implying that manipulation of the transporters involved in citrate uptake and export would be a desirable strategy for increasing the rate of citrate production.

Since the biosynthesis of itaconic acid in *A. terreus* is very similar to that of citric acid in *A. niger* in some aspects, the same theory might be applicable to itaconate production by *A. terreus* and help to explain some above results. Respective overexpression of the central carbon metabolism genes such as mt-*pfkA*, *gpdA*, *citA* and *acoA* didn’t increase itaconate productivity, while over-expression of *mfsA*, the potential itaconate exporter, led to the enhanced itaconate production level. However, overexpression of *cadA*, resulting in the enhanced itaconate titer, couldn’t be explained by this theory. Thus, a different theory may be required for itaconic acid production.

## Conclusions

In conclusion, the genes which are closely related to itaconate production were identified and overexpressed in an industrially used *A. terreus* strain, including *cadA*, *mfsA*, *mttA*, ATEG_09969, *gpdA*, ATEG_01954, *acoA*, *citA* and mt*-pfk*A. Overexpression of *cadA* and *mfsA* enhanced the itaconate production level, and introduction of other genes showed varied effects on itaconic acid production. Extracellular organic acids rather than itaconic acid were affected by overexpression of these genes to different extent. The genetic engineering approach will be useful for increasing itaconate production capacity and lowering the cost of industrial production.

## Methods

### Materials

Chemicals were obtained from Sigma, Merck or Ameresco. Oligonucleotides were synthesized by Shanghai Sangon Biotech Co. Ltd (China). *Taq* and *pfu* DNA polymerases, RevertAid Reverse Transcriptase, restriction endonucleases were purchased from Fermentas or New England BioLabs. The kits used for molecular cloning were bought from Omega Bio-tek Biotechnology. Difco™Potato Dextrose Agar was from BD. Trizol was from Invitrogen Life Technologies Corporation. DIG High Prime DNA Labelling and Detection Starter Kit I was purchased from RocheApplied Science. Hygromycin B was obtained from Solarbio Science Technology Co., Ltd (China).

### Plasmids, strains, media and cultivation conditions

The plasmids pAN52-4 and pAN7-1 were kindly provided by Professor Punt from TNO Microbiology and Systems Biology (Netherlands) [24,42]. Spores were harvested from 7-day-old potato dextrose agar plate (PDA). Cultivation in shake flasks was carried out in 500 ml shake flasks containing 55 ml itaconic acid production medium (IPM) on a rotary shaker at 200 rpm and 37°C [10].

### DNA manipulations and transformation

General molecular biology techniques were carried out following the standard procedures [43]. The primers for the target genes from *A. terreus* were designed on the basis of the genome sequence of *A. terreus* NIH 2624 (http://www.broadinstitute.org/annotation/genome/aspergillus_group/FeatureSearch.html). All primers were listed in Table S1 (Additional file 7).

The DNA fragments of *mttA* (ATEG_09970), *cadA* (ATEG_07991), ATEG_09969, *gpdA* (ATEG_09817), *acoA* (ATEG_03325) and *citA* (ATEG_07990) were amplified from the cDNA of *A. terreus* LYT10 using the respective primer pairs. The *mfsA* gene was cloned as follows: The sequence of the cloned *mfsA* fragment is slightly different from the annotated one at the 3’ end when amplified using the primers *mfsA*-F and *mfsA*-R1. The primer *mfsA*-L-R1 was then designed at about 500 bp downstream of the annotated stop codon of *mfsA*, and the DNA fragment containing *mfsA* was amplified from the genomic DNA of *A. terreus* LYT10. Based on the sequencing result, a series ofnew reverse primers (*mfsA*-L-R2, *mfsA*-L-R3,*mfsA*-L-R4,*mfsA*-L-R5) at the downstream of the annotated stop codon of *mfsA* were redesigned to clone the *mfsA* gene from the cDNA of *A. terreus* LYT10.The DNA fragment amplified using the primers *mfsA*-F and *mfsA*-L-R2 covers the full ORF of the *mfsA* gene. The new introns and stop codon were identified, and the new sequence has been deposited with GenBank Accession No. KF305087. Finally, *mfsA*was amplified using the primers *mfsA*-F and *mfsA*-R2 for further subcloning. The sequence identity between the cDNAs of *mfsA* from *A. terreus* LYT10 and *A. terreus* NIH2624 is 80.5% (Additional file 8: Figure S7). The sequence identity between them is 81.5% at the protein level (Additional file 9: Figure S8). The difference is possibly due to annotation of the introns and stop codon for *mfsA*.

Truncated *pfkA* in which 335 amino acids were removed at the C-terminus of PFK1 was amplified from the cDNA of *A. niger* Co827, and the phosphorylation site Thr 89 of *pfkA* was mutated using the primers mt-*pfkA*-F/mt-*pfkA*-R as reported [23].

All DNA fragments were inserted at the restriction sites of *Nco*I and *Bam*HI or *Hind*III of the vector pAN52-4 to obtain the pAN52-XX (XX, the different genes) plasmids respectively. The linearized plasmids by *Dra*I and *Eco*RI were co-transformed with pAN7-1 into *A. terreus* LYT10 respectively [14]. Transformants were selected on the PDA-SH plates (PDA supplemented with 1.2 M sorbitol and 100 mg/L hygromycin B). The integrations of the target genes into the genome were confirmed by genomic PCR using the primers pAN-seq-F and pAN-seq-R. Five transformants for each gene were randomly selected for genomic PCR.

ATEG_01954 was amplified from the cDNA of *A. terreus* LYT10 and cloned into the vector pG3H at the restriction sites of *Pci*I and *Bgl*II [25]. The linearized plasmid was transformed into *A. terreus* LYT10. Transformants were selected on the PDA-SH plates. The integration of ATEG_01954 into the genome was confirmed by genomic PCR using the primers M13-47 and ATEG_01954-R1.Five transformants were randomly selected for genomic PCR.

The *hph* cassette was cut from the pSGF957 plasmid with *Xba*I and inserted into pAN52-*cadA* digested with *Xba*I to get pAN52-*cadA*-*hph*. The linearized plasmids pAN52-*cadA*-*hph* and pAN52-*mfsA* were co-transformed into *A. terreus* LYT10. The co-integrations of the *cadA* and *mfsA* gene were confirmed by genomic PCR using the primers pAN-seq-F and cadA-R or mfsA-R.

Southern blot analysis was performed using DIG High Prime DNA Labelling and Detection Starter Kit I (Roche Applied Science) according to the manufacturer’s instructions. Genomic DNAs of the selected *cadA*- and *mfsA*-transformants (*cadA*-2, −5, −18, −21, −22, and *mfsA*-2, −10, −17, −12, −24) were isolated by Fungal DNA Kit (Omega Bio-tek) and digested with *BamH*I at 37°C overnight. The DNA probes of *cadA* and *mfsA* were amplified by PCR using the primer pairs CADt-F/CADt-R and MFSt-F/MFSt-R respectively.

### Analysis of the transformants

The transformants, which can grow healthily on the PDA plates, were tested for itaconic acid production in 50 ml IPM on a rotary shaker at 37°C for 76 h. Each transformant was grown in three individual flaks, and the screening experiments for the important transformants were repeated once. The titers of itaconic acid produced were determined by HPLC (High Performance Liquid Chromatography) using an Aminex HPX-87-H column (300 mm × 7.8 mm) detected at 210 nm. The column was operated at 35°C with a mobile phase of 4 mM H_2_SO_4_ at a flow rate of 0.6 ml/min. Authentic itaconic acid was used as a standard to calculate the final concentration of itaconic acid.

### Analysis of extracellular organic acids

The concentrations of extracellular organic acids (itaconic, citric, *cis*-aconitic, pyruvic, malic, α-ketoglutaric, lactic succinic, fumaric and oxalic acid) in the samples were determined by HPLC. Organic acids were separated on a Bio-Rad HPX-87H column by HPLC at 35°C, using 5 mM H_2_SO_4_ as the mobile phase at a flow rate of 0.6 mL min^−1^ and detected by a refractive index detector and a UV–vis one (at 210 nm). The standard curves of all organic acids were established using 0.5 mM crotonic acid as an internal standard.

### Itaconic acid production by *cadA*-21 at the demonstration scale

The demonstration experiment by *cadA*-21was performed in IPM using a 35 m^3^ fermentor. A two-stage process including the vegetative phase and the production phase was used [40], and the vegetative phase was done for 16–18 hr in 4 m^3^ fermentor. The only difference between the vegetative medium and IPM is glucose concentration: 40 g/L *versus* 130 g/L. The fermentation stage was carried out with the following conditions: 37°C, 250 rev/min, 1 × 10^7^ spores/mL (the initial spore concentration), and 20 m^3^/min (aeration). Samples were taken at regular intervals for analysis. Residual glucose was quantified using a biosensor (SBA-40C) from Biology Institute of Shandong Academy of Science (Jinan, China) following the standard protocol. The titer of produced itaconic acid was quantified by HPLC as above.

## Additional files

Additional file 1: Figure S1. Genomic PCR analysis of the randomly selected transformants of each gene. Additional file 2: Figure S2. Southern blot analysis of the *cadA* (A) - and *mfsA* (B) –transformants. Additional file 3: Figure S3. Itaconic acid production by *cadA* (A) and *mfsA* (B) transformants. *A. terreus* LYT10 was as a reference (WT). The transformants were screened for itaconate production, and the itaonate titers after 76-hr incubation were determined by HPLC. Additional file 4: Figure S4. Itaconic acid production by co-transformant of *cadA* and *mfsA. A. terreus* LYT10 was used as a reference (WT). Thetransformants were screened for itaconate production, and the itaonate titers after 76-hr incubation were determined by HPLC. Additional file 5: Figure S5. HPLC analysis of itaconic acid produced by the best transformant *cadA*-21 (A, C) and *A. terreus* LYT10 (B, D). Samples were analyzed by HPLC after 76-hr incubation. A, C: the whole chromatograms; B, D: the enlarged ones for small peaks. Itaconic acid: the peak at 13.3 min. Additional file 6: Figure S6. Time courses of residual glucose (A) and itaconate production (B) for WT and strain *cadA*-21 at the pilot scale. The demonstration experiment was performed in a 35 m^3^ fermentor. A two-stage process including the vegetative phase and the production phase was applied, and the vegetative phase was done for 16–18 hr in 4 m^3^ fermentor. Samples were taken at regular intervals for analysis. In the *inset*, changes of glucose concentrations and itaconate titers during the final period were expanded. Additional file 7: Table S1. The primers used in this study. Additional file 8: Figure S7. Sequence alignment of *mfsA*-DNA-LYT10 (top), *mfsA*-cDNA-LYT10 (middle), and *mfsA*-cDNA-NIH2624 (bottom) LYT10, *A. terreus* LYT10; NIH2624, *A. terreus* NIH2624. Additional file 9: Figure S8. Sequence alignment of MFS from *A. terreus* NIH 2624 (up) and *A. terreus* LYT10 (down) (at the protein level).

## Abbreviations

ACO: Aconitase
CAD: *cis*-aconitate decarboxylase
CS: citrate synthase
IPM: itaconate production medium
MFS: Major Facilitator Superfamily
MTT: Mitochondrial tricarboxylic transporter
