# Supplementary material for: Improving itaconic acid production through genetic engineering of an industrial Aspergillus terreus strain
Source: Microb Cell Fact. 2014 Aug 11;13:119. doi: 10.1186/s12934-014-0119-y (PMC4251695; doi:10.1186/s12934-014-0119-y)

Additional file 1

Figure S1 Genomic PCR analysis of randomly selected transformants of each gene. Five transformants were randomly selected for genomic PCR. The integrations of most target genes into the genome were confirmed by PCR using the primers pAN-seq-F and pAN-seq-R, whereas the integration of ATEG_01954 was confirmed using the primers M13-47 and ATEG_01954-R1, and the co-integrations of the *cadA* and *mfsA* gene were confirmed using the primers pAN-seq-F and cadA-R or mfsA-R. In most cases, both the target genes and *hph* were amplified except co-transformants of *cadA* and *mfsA* (**E**) and transformants of ATEG_01954 (**G**). The target genes were detected in most transformants.

A: *mttA*-transformants

Lane 1, 1 kb DNA ladder; Lanes 2-7, transformants 2, 5, 6, 7, 12, 16; Lane 8, WT; Lane 9, pAN7-1.

B: *cadA*-transformants

Lane 1, 1 kb DNA ladder; Lanes 2-7, transformants 2, 5, 12, 18, 21, 22; Lane 8, WT.

C: *mfsA*-transformants

Lane 1, 1 kb DNA ladder; Lanes 2-7, transformants 2, 9, 10, 12, 17, 24; Lane 8, WT.

D: ATEG_09969-transformants

Lane 1, 1 kb DNA ladder; Lanes 2-7, transformants 4, 5, 6, 9, 10, 12.

E: co-transformants of *cadA* and *mfsA*

Lane 1, 1 kb DNA ladder; Lanes 2-8, the *cadA* fragment amplified from transformants 3, 4, 5, 6, 7 ,8 , 9; Lane 9, WT; Lane 10, DL200 DNA ladder; Lanes 11-17, the *mttA* fragment amplified from transformants 3, 4, 5, 6, 7 ,8 , 9; Lane 18, WT.

F: *gpdA*-transformants

Lane 1, 1 kb DNA ladder; Lanes 2-7, transformants 1, 4, 7, 12, 23, 24; Lane 8, WT.

G: ATEG_01954-transformants

Lane 1, DL 2000 DNA ladder; Lanes 2-7, transformants 1, 3, 6, 7, 11, 13; Lane 8, WT.

H: *acoA*-transformants

Lane 1, 1 kb DNA ladder; Lanes 2-7, transformants 1, 5, 7, 9, 14, 23; Lane 8, WT.

I: *mt-pfkA*-transformants

Lane 1, 1 kb DNA ladder; Lanes 2-7, transformants 3, 8, 13,15, 16,19; Lane 8, WT; Lane 9, pAN7-1; Lane 10, pAN52-mtpfkA.

J: *citA*-transformants

Lane 1, 1 kb DNA ladder; Lanes 2-7, transformants 6, 7, 12, 21, 22, 23; Lane 8, WT.

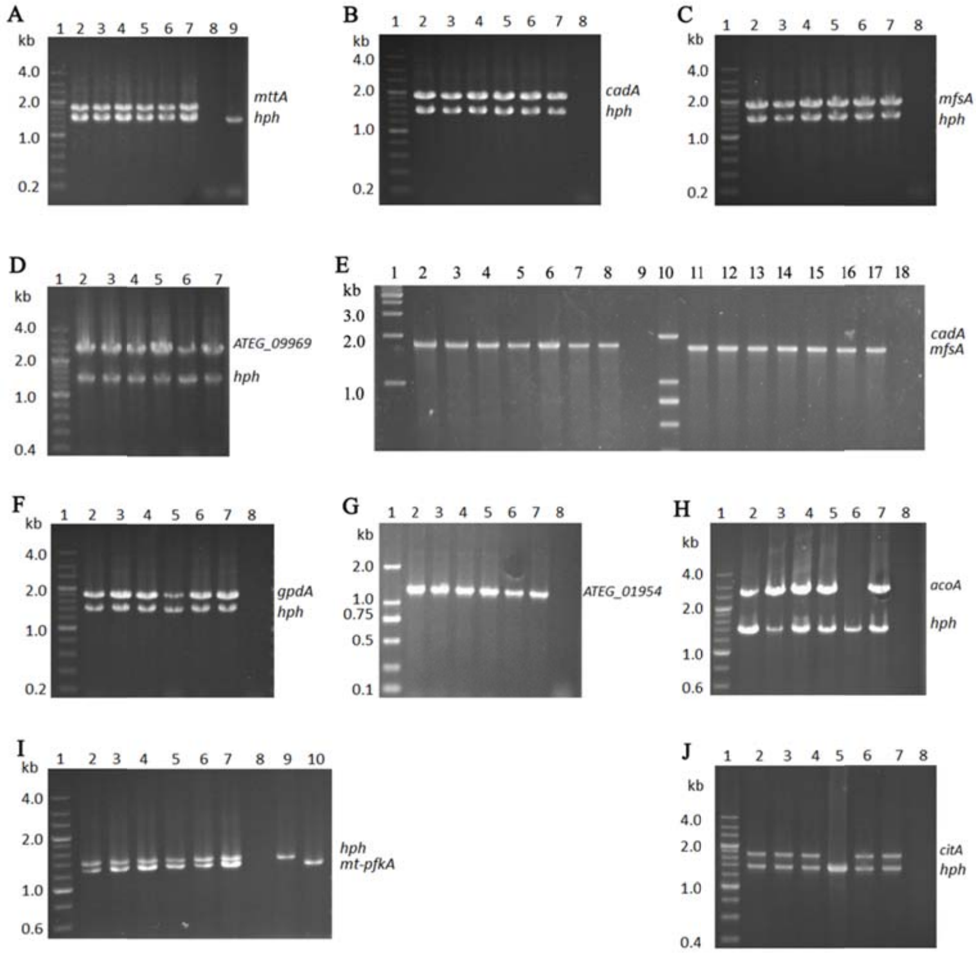

Supplement: Additional file 1: Figure S1. — Genomic PCR analysis of the randomly selected transformants of each gene. [file 12934_2014_119_MOESM1_ESM.pdf]
