# Supplementary material for: Improving itaconic acid production through genetic engineering of an industrial Aspergillus terreus strain
Source: Microb Cell Fact. 2014 Aug 11;13:119. doi: 10.1186/s12934-014-0119-y (PMC4251695; doi:10.1186/s12934-014-0119-y)
Supplement: Additional file 2: Figure S2. — Southern blot analysis of the cadA (A) - and mfsA (B) –transformants. [file 12934_2014_119_MOESM2_ESM.pdf]

Additional file 2

Figure S2 Southern blot analysis of *cadA* (A) - and *mfsA* (B) –transformants. Southern blot analysis was performed using DIG High Prime DNA Labelling and Detection Starter Kit I (Roche Applied Science) according to the manufacturer's instructions. Genomic DNAs of selected *cadA*- and *mfsA*-transformants were digested with *Bam*HI at 37°C overnight. The DNA probes of *cadA* and *mfsA* were amplified by PCR using the primer pairs CADt-F/CADt-R and MFSt-F/MFSt-R respectively.

Generally, one band corresponds to one copy. However, for the samples of strains *cadA*-5 and *mfsA*-12, two bands are much stronger than others in the same sample, so two copies were considered as corresponding to these two bands. 2-, 4-, 3-, 1-, and 2-copy of the *cadA* genes were integrated in transformants*cadA*-2, -5, -18, -21, and -22 respectively (**A**), whereas 1-, 4-, 4-, 8-, and 2-copy of the *mfsA* genes were present in transformants*mfsA*-2, -10, -17, -12, and -24 respectively (**B**).

A: Lane 1, Marker; Lane 2, *cadA*-2 transformant; Lane 3, *cadA*-5 transformant; Lane 4, *cadA*-18 transformant; Lane 5, *cadA*-21 transformant; Lane 6, *cadA*-22 transformant; Lane 7, WT.

B: Lane 1, Marker; Lane 2, *mfsA*-2 transformant; Lane 3, *mfsA*-10 transformant; Lane 4, *mfsA*-17 transformant; Lane 5, *mfsA*-12 transformant; Lane 6, *mfsA*-24 transformant; Lane 7, WT.

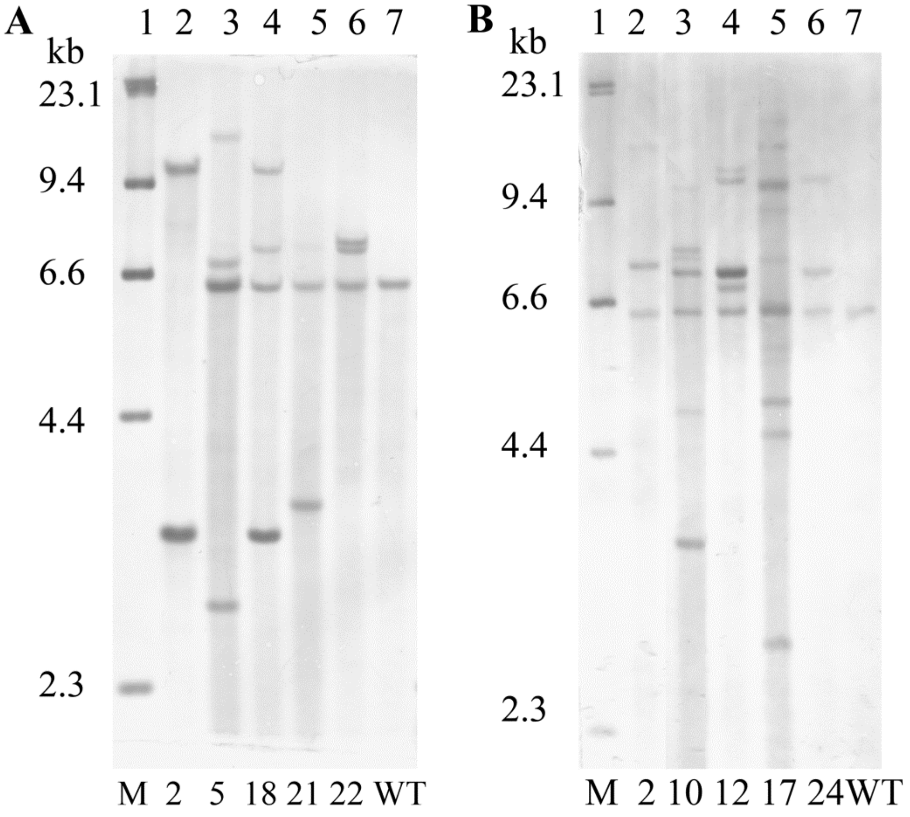
A
kb
1 2 3 4 5 6 7
23.1
9.4
6.6
4.4
2.3
M 2 5 18 21 22 WT
B
kb
1 2 3 4 5 6 7
23.1
9.4
6.6
4.4
2.3
M 2 10 12 17 24 WT
