# Supplementary material for: Improving itaconic acid production through genetic engineering of an industrial Aspergillus terreus strain
Source: Microb Cell Fact. 2014 Aug 11;13:119. doi: 10.1186/s12934-014-0119-y (PMC4251695; doi:10.1186/s12934-014-0119-y)

Additional file 3

Figure S3 Itaconic acid production by *cadA* (A) and *mfsA* (B) transformants. *A. terreus* LYT10 was as a reference (WT)

The transformants were screened for itaconate production, and the itaonate titers after 76-hr incubation were determined by HPLC.

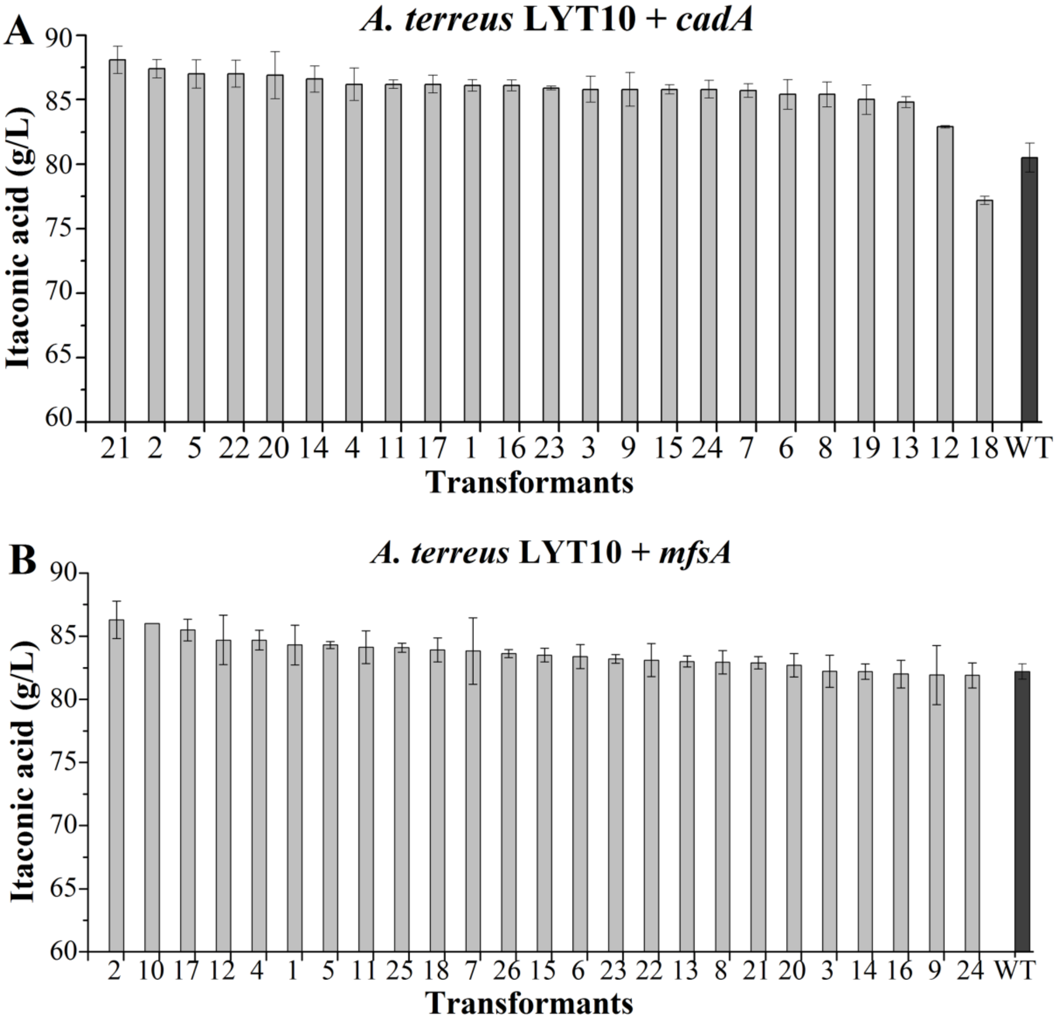

Supplement: Additional file 3: Figure S3. — Itaconic acid production by cadA (A) and mfsA (B) transformants. A. terreus LYT10 was as a reference (WT). The transformants were screened for itaconate production, and the itaonate titers after 76-hr incubation were determined by HPLC. [file 12934_2014_119_MOESM3_ESM.pdf]
