# Supplementary material for: Improving itaconic acid production through genetic engineering of an industrial Aspergillus terreus strain
Source: Microb Cell Fact. 2014 Aug 11;13:119. doi: 10.1186/s12934-014-0119-y (PMC4251695; doi:10.1186/s12934-014-0119-y)

Additional file 4

Figure S4 Itaconic acid production by co-transformant of *cadA* and *mfsA*. *A. terreus* LYT10 was used as a reference (WT)

The transformants were screened for itaconate production, and the itaonate titers after 76-hr incubation were determined by HPLC.

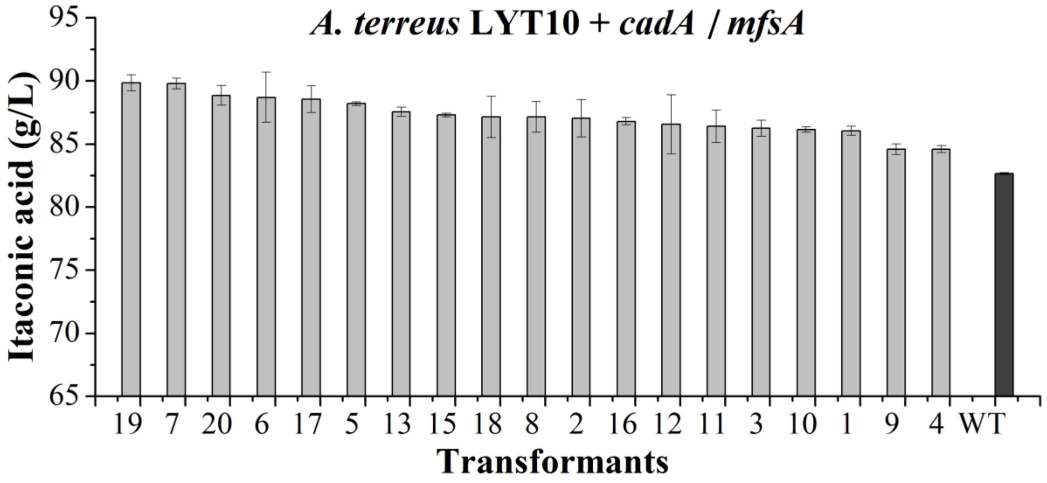

Supplement: Additional file 4: Figure S4. — Itaconic acid production by co-transformant of cadA and mfsA. A. terreus LYT10 was used as a reference (WT). Thetransformants were screened for itaconate production, and the itaonate titers after 76-hr incubation were determined by HPLC. [file 12934_2014_119_MOESM4_ESM.pdf]
