# Supplementary material for: Improving itaconic acid production through genetic engineering of an industrial Aspergillus terreus strain
Source: Microb Cell Fact. 2014 Aug 11;13:119. doi: 10.1186/s12934-014-0119-y (PMC4251695; doi:10.1186/s12934-014-0119-y)
Supplement: Additional file 5: Figure S5. — HPLC analysis of itaconic acid produced by the best transformant cadA-21 (A, C) and A. terreus LYT10 (B, D). Samples were analyzed by HPLC after 76-hr incubation. A, C: the whole chromatograms; B, D: the enlarged ones for small peaks. Itaconic acid: the peak at 13.3 min. [file 12934_2014_119_MOESM5_ESM.pdf]

Additional file 5

Figure S5 HPLC analysis of itaconic acid produced by the best transformant *cadA*-21 (A, C) and *A. terreus* LYT10 (B, D)

Samples were analyzed by HPLC after 76-hr incubation**. A**, **C**: the whole chromatograms; **B**, **D**: the enlarged ones for small peaks. Itaconic acid: the peak at 13.3 min.

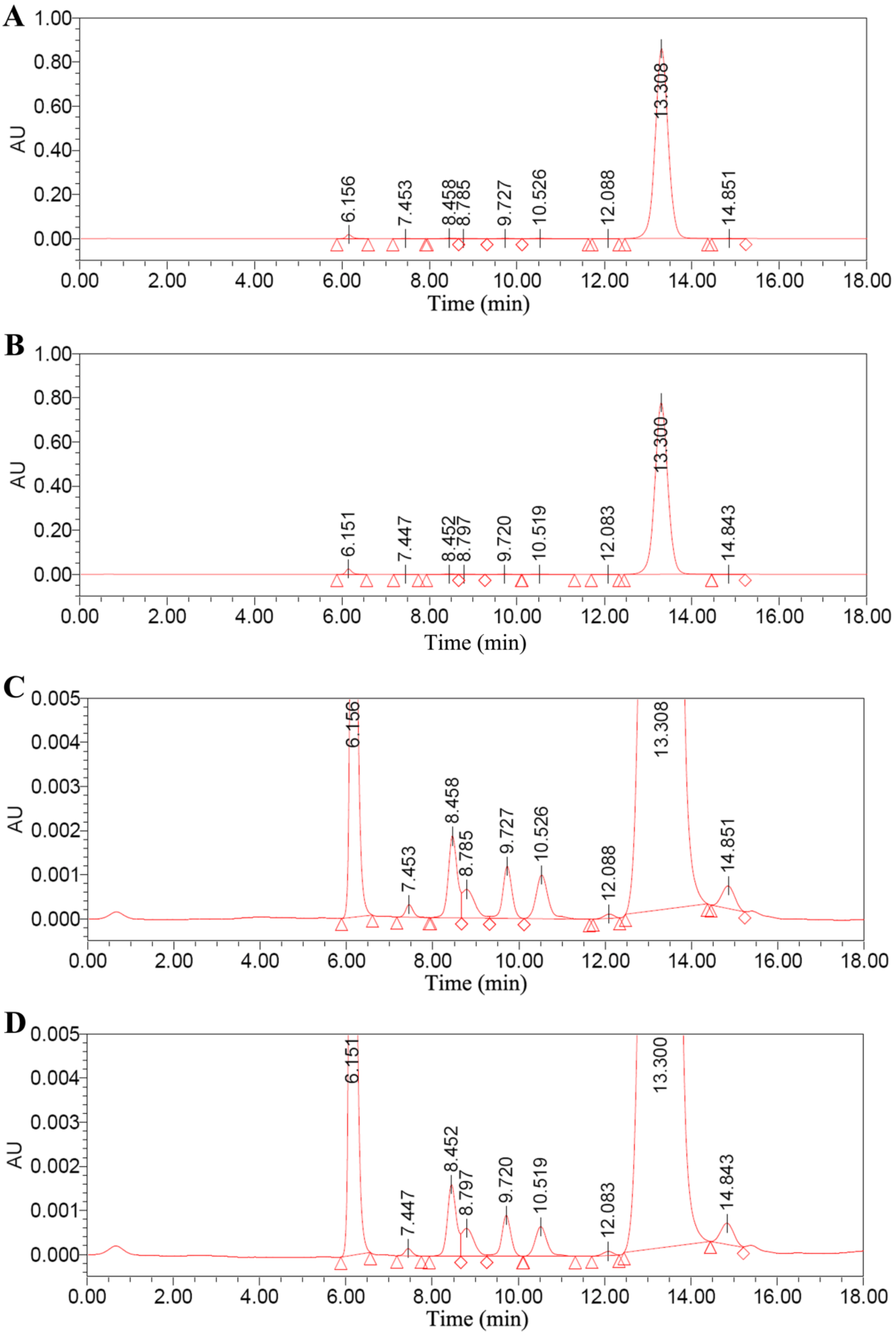
A
B
C
D
AU
Time (min)
1.00
0.80
0.60
0.40
0.20
0.00
0.005
0.004
0.003
0.002
0.001
0.000
0.00
2.00
4.00
6.00
8.00
10.00
12.00
14.00
16.00
18.00
6.156
7.453
8.458
8.785
9.727
10.526
12.088
13.308
14.851
6.151
7.447
8.452
8.797
9.720
10.519
12.083
13.300
14.843
