# Supplementary material for: Improving itaconic acid production through genetic engineering of an industrial Aspergillus terreus strain
Source: Microb Cell Fact. 2014 Aug 11;13:119. doi: 10.1186/s12934-014-0119-y (PMC4251695; doi:10.1186/s12934-014-0119-y)

Additional file 6

Figure S6 Time courses of residual glucose (A) and itaconate production (B) for WT and strain *cadA*-21 at the pilot scale.

The demonstration experiment was performed in a 35 m^3 fermentor. A two-stage process including the vegetative phase and the production phase was applied, and the vegetative phase was done for 16-18 hr in 4 m^3 fermentor. Samples were taken at regular intervals for analysis. In the *inset*, changes of glucose concentrations and itaconate titers during the final period were expanded.

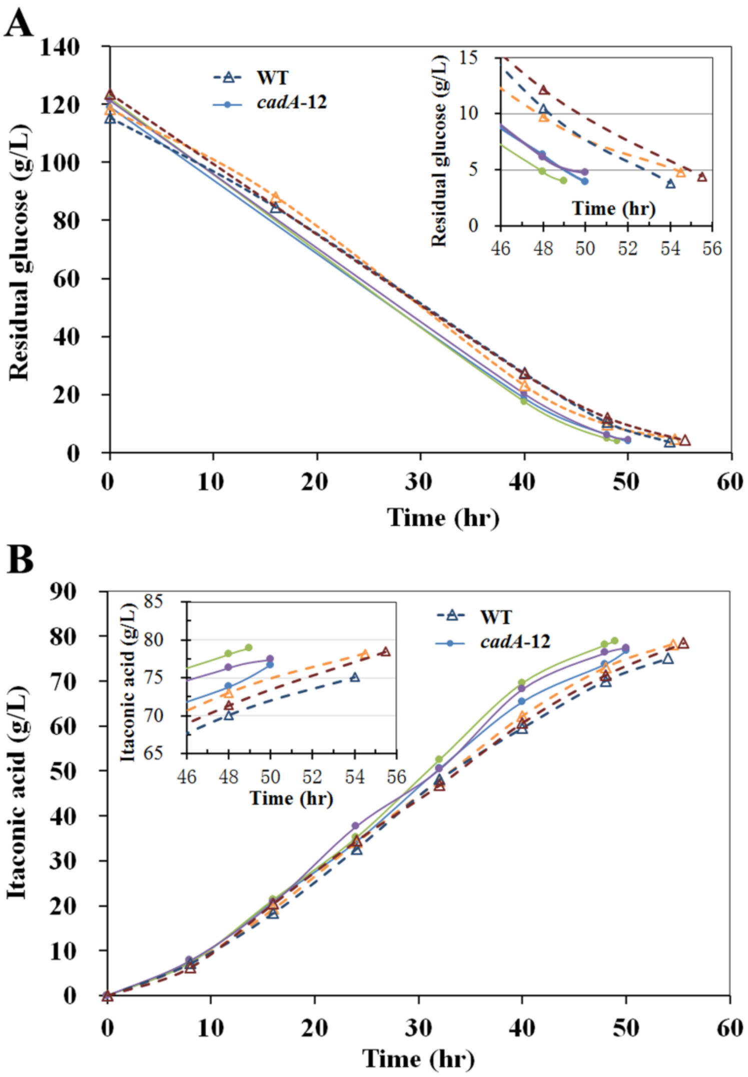

Supplement: Additional file 6: Figure S6. — Time courses of residual glucose (A) and itaconate production (B) for WT and strain cadA-21 at the pilot scale. The demonstration experiment was performed in a 35 m3 fermentor. A two-stage process including the vegetative phase and the production phase was applied, and the vegetative phase was done for 16–18 hr in 4 m3 fermentor. Samples were taken at regular intervals for analysis. In the inset, changes of glucose concentrations and itaconate titers during the final period were expanded. [file 12934_2014_119_MOESM6_ESM.pdf]
