# Supplementary material for: Improving itaconic acid production through genetic engineering of an industrial Aspergillus terreus strain
Source: Microb Cell Fact. 2014 Aug 11;13:119. doi: 10.1186/s12934-014-0119-y (PMC4251695; doi:10.1186/s12934-014-0119-y)
Supplement: Additional file 7: Table S1. — The primers used in this study. [file 12934_2014_119_MOESM7_ESM.pdf]

Additional file 7

Table S1The primers used in this study

Primers	Sequence 5'→3'(Restriction sites underlined)
cadA-F	GCATTCATGACCAAACAATCTGCGGACAGC (*Bsp*HI)
c*adA*-R	GGCGGATCCTTATACCAGTGGCGATTTCACGG (*Bam*HI)
pfkA-F	GATCATGAGTGCTCCCCCCCAAGCTC (*Bsp*HI)
pfkA-R	TGGATCCTTACCCGGGATCATAGTGCCGGCACAGACC (*Bam*HI)
mt-*pfkA*-F	CCGCCCGCTGCATGGAGTTCCGTGAGCGCCC
mt-*pfkA*-F	GGGCGCTCACGGAACTCCATGCAGCGGGCGG
gpdA-F3	GTGCCATGGCTCCCAAGGTCGGTATC (*Nco*I)
gpdA-R	CATGGATCCCTACTGGGCATCAACCTTGGAG (*Bam*HI)
mttA-F2	GTGCCATGGACTCTAAAATCCAGACAAAT (*Nco*I)
mttA-R	GGCAAGCTTTCAGTTTGGTTGCGTCAAGAAC (*Hind*III)
citA-F2	GCATTCATGATGGCTGCCTCTCTCAGACTCGGAAC (*Bsp*HI)
citA-R	CAT GGATCCTTACAGCTTAGCGCCAACCAG (*Bam*HI)
ATEG_09969-F	GTGCCATGGAGAGTGCAGAGCTGTC (*Nco*I)
ATEG_09969-R	GGCAAGCTTTTAAACAGGCCTATCTAGTAGTCTC (*Hind*III)
acoA-F1	CATTCATGATCTCCACCCGCCTTGC (*Bsp*HI)
acoA-R1	CATAGATCTTAGTTGCTAGCAGCCTTGCGG (*Bgl*II)
ATEG_01954-F1	GCATTCATGAAGCTCAATCTCCTCGCTGTC (*Bsp*HI)
ATEG_01954-R1	CATAGATCTTACAGAGCCCAGATGCCCAGAG (*Bgl*II)
mfsA-F	GCATTCATGAGCCACGGTGACACTGAGTCC (*Bsp*HI)
mfsA-R1	GGCGGATCCTTATTGTGGGCATGTACACAAAGG (*Bam*HI)
mfsA-L-R1	TAGACCTGGCTGTAAGCAG

mfsA-L-R2	CCCGCATCCGATGAATTGTTGACG
mfsA-R2	GGCGGATCCTTATTGGGTAGGCATATTGAAAGC (*Bam*HI)
pAN-seq-F	GTCGTTGCGTCAGTCCAACATTTG
pAN-seq-R	CGAGATCCTGAACACCATTTGTCTC
CADt-F	GAGGCTATCGAAAACCTTC
CADt-R	CACCAAGAGGCTTCTCGAC
MFSt-F	GCCACGGTGACACTGAGTC
MFSt-R	TCTGCAAGAACAGTTTGGC
