# Supplementary material for: Improving itaconic acid production through genetic engineering of an industrial Aspergillus terreus strain
Source: Microb Cell Fact. 2014 Aug 11;13:119. doi: 10.1186/s12934-014-0119-y (PMC4251695; doi:10.1186/s12934-014-0119-y)
Supplement: Additional file 8: Figure S7. — Sequence alignment of mfsA-DNA-LYT10 (top), mfsA-cDNA-LYT10 (middle), and mfsA-cDNA-NIH2624 (bottom) LYT10, A. terreus LYT10; NIH2624, A. terreus NIH2624. [file 12934_2014_119_MOESM8_ESM.pdf]

Additional file 8

Figure S7 Sequence alignment of *mfsA*-DNA-LYT10 (top), *mfsA*-cDNA-LYT10 (middle), and *mfsA*-cDNA-NIH2624 (bottom)

```
                  1                                                         60
mfsA-DNA-LYT10    ATGAGCCACGGTGACACTGAGTCCCCAAACCCAGTGACATCCACGGAAGGTAGCGGGCAA
mfsA-cDNA-LYT10   ATGGGCCACGGTGACACTGAGTCCCCAAACCCAGTGACATCCACGGAAGGTAGCGGGCAA
mfsA-cDNA-NIH2624 ATGGGCCACGGTGACACTGAGTCCCCGAACCCAACGACGACCACGGAAGGTAGCGGACAA
                  *** ********************** ******  ***  **************** ***
                  61                                                       120
mfsA-DNA-LYT10    TCTGAGCCAGAGAAAAGGGGCCCTGATATTCCACTATGGAGAAAATGCGTCATTACGTTT
mfsA-cDNA-LYT10   TCTGAGCCAGAGAAAAGGGGCCCTGATATTCCACTATGGAGAAAATGCGTCATTACGTTT
mfsA-cDNA-NIH2624 AACGAGCCAGAGAAAAAGGGCCGTGATATTCCATTATGGAGAAAATGTGTCATTACGTTT
                     ************* ***** ********** ************* ************
                  121                                                      180
mfsA-DNA-LYT10    GTTGTTAGTTGGATGACTCTAGTCGTTACTTTCTCCAGTACTTGCCTTCTTCCTGCCGCC
mfsA-cDNA-LYT10   GTTGTTAGTTGGATGACTCTAGTCGTTACTTTCTCCAGTACTTGCCTTCTTCCTGCCGCC
mfsA-cDNA-NIH2624 GTTGTTAGTTGGATGACTCTAGTCGTTACTTTCTCCAGTACTTGTCTTCTTCCTGCCGCC
                  ******************************************** ***************
                  181                                                      240
mfsA-DNA-LYT10    CCTGAAATTGCGGGTGAATTTGATATGACTGTCGAGACTATCAATATCTCCAATGCTGGT
mfsA-cDNA-LYT10   CCTGAAATTGCGGGTGAATTTGATATGACTGTCGAGACTATCAATATCTCCAATGCTGGT
mfsA-cDNA-NIH2624 CCTGAAATCGCGAATGAATTTGATATGACTGTCGAGACTATCAACATCTCCAATGCTGGT
                  ******** ***  ****************************** ***************
                  241                                                      300
mfsA-DNA-LYT10    GTCCTGATTGCCATGGGATATTCTTCCCTCATATGGGGTCCCATGAACAAGTTAATCGGC
mfsA-cDNA-LYT10   GTCCTGATTGCCATGGGATATTCTTCCCTCATATGGGGTCCCATGAACAAGTTAATCGGC
mfsA-cDNA-NIH2624 GTCCTAGTTGCCATGGGATATTCATCCCTCATATGGGGTCCCATGAACAAGTTAGTCGGC
                  *****  **************** ****************************** *****
                  301                                                      360
mfsA-DNA-LYT10    AGGCGGACATCATACAATCTGGCCATTTCAATGCTTTGTGCGTGCTCCGCTGGAACGGCA
mfsA-cDNA-LYT10   AGGCGGACATCATACAATCTGGCCATTTCAATGCTTTGTGCGTGCTCCGCTGGAACGGCA
mfsA-cDNA-NIH2624 CGGCGGACATCATACAATCTGGCCATTTCAATGCTTTGTGCATGCTCCGCTGGAACGGCA
                   **************************************** ******************
                  361                                                      420
mfsA-DNA-LYT10    GCGGCGATAAACGAGGAAATGTTCATAGCGTTCAGAGTGTTGAGCGGCTTAACCGGAACC
mfsA-cDNA-LYT10   GCGGCGATAAACGAGGAAATGTTCATAGCGTTCAGAGTGTTGAGCGGCTTAACCGGAACC
mfsA-cDNA-NIH2624 GCGGCGATAAACGAGGAAATGTTCATAGCGTTCAGAGTGTTGAGCGGCTTAACCGGAACC
                  ************************************************************
                  421                                                      480
mfsA-DNA-LYT10    TCGTTCATGGTCTCAGGCCAAACTGTTCTTGCAGACATCTTTGAGCCTGTACGCATAACA
mfsA-cDNA-LYT10   TCGTTCATGGTCTCAGGCCAAACTGTTCTTGCAGACATCTTTGAGCCTGT----------
mfsA-cDNA-NIH2624 TCGTTCATGGTCTCAGGCCAAACTGTTCTTGCAGATATCTTTGAGCCTGT----------
                  *********************************** **************
                  481                                                      540
mfsA-DNA-LYT10    CGCCCTCGTCACCCTATTTTCGAAAACTAATCTGTTCGTGTGCAGGTTTACCGTGGGACG
mfsA-cDNA-LYT10   -----------------------------------------------TTACCGTGGGACG
mfsA-cDNA-NIH2624 -----------------------------------------------TTACCGTGGGACG
                                                                 *************
                  541                                                      600
mfsA-DNA-LYT10    GCCGTAGGTTTCTTCATGGCTGGGACTCTTTCTGGCCCTGCAATAGGTACATCCTCGGCG
mfsA-cDNA-LYT10   GCCGTAGGTTTCTTCATGGCTGGGACTCTTTCTGGCCCTGCAATAGG-------------
mfsA-cDNA-NIH2624 GCCGTAGGTTTCTTCATGGCCGGGACTCTTTCTGGCCCTGCAATAGG-------------
                  ******************** **************************
                  601                                                      660
mfsA-DNA-LYT10    GCAAGTACTAGAACTCCGAACAGGAACTAATTGTTTGACCAGGCCCGTGCGTGGGAGGGA
mfsA-cDNA-LYT10   -------------------------------------------CCCGTGCGTGGGAGGGA
mfsA-cDNA-NIH2624 -------------------------------------------CCCCTGCGTGGGAGGGG
                                                             *** ************
                  661                                                      720
mfsA-DNA-LYT10    TCATCGTCACTTTCACGAGTTGGCGTGTTATCTTCTGGCTCCAACTAGCTATGAGCGGGC
mfsA-cDNA-LYT10   TCATCGTCACTTTCACGAGTTGGCGTGTTATCTTCTGGCTCCAACTAGCTATGAGCGGGC
mfsA-cDNA-NIH2624 TCATCGTCACTTTCACGAGTTGGCGTGTTATCTTCTGGCTTCAACTAGGTATGAGCGGGC
                  **************************************** ******* ***********
                  721                                                      780
mfsA-DNA-LYT10    TGGGGCTAGTTCTTTCTCTTCTATTTTTCCCGAAAGTCGAAGCACTTTCTGAGAAGGCGT
mfsA-cDNA-LYT10   TGGGGCTAGTTCTTTCTCTTCTATTTTTCCCGAAAGTCGAAGCACCTTCTGAGAAGGCCT
mfsA-cDNA-NIH2624 TGGGGCTCGTGCTTTCTCTGCTATTTTTCCCGAAAATCGAAGGAAATTCTGAGAAGGTCT
                  ******* ** ******** *************** ****** *  ***********  *
                  781                                                      840
mfsA-DNA-LYT10    CAACCGCGTCCAAACCGACCACACTTGTCACAATCATATCAAAATTCTCCCCAACGGATG
mfsA-cDNA-LYT10   CAACCGCGTCCAAACCGACCACACTTGTCACAATCATATCAAAATTCTCCCCAACGGATG
mfsA-cDNA-NIH2624 CAACGGCGTTTAAACCGACCACACTTGTCACAATCATATCGAAATTCTCCCCAACGGATG
                  **** ****  ***************************** *******************
                  841                                                      900
mfsA-DNA-LYT10    TGCTCAAGCAGTGGGTCTATCCAAATATCTTTCTTGCCGTAAGTATCTGCGAGATATACC
mfsA-cDNA-LYT10   TGCTCAAGCAGTGGGTCTATCCAAATATCTTTCTTGCCG---------------------
mfsA-cDNA-NIH2624 TGCTCAAGCAGTGGGTGTATCCAAATGTCTTTCTTGCCG---------------------
                  **************** ********* ************
```

```
                  901                                                        960
mfsA-DNA-LYT10    CTATGCATTTACTGGAAAACAAAATGCTCATGCCGCAAATCAAAGGACTTATGCTGTGGC
mfsA-cDNA-LYT10   ----------------------------------------------ACTTATGCTGTGGC
mfsA-cDNA-NIH2624 ----------------------------------------------ACTTATGCTGTGGC
                                                                **************
                  961                                                       1020
mfsA-DNA-LYT10    CTCCTGGCGATTACGCAATATTCGATCCTGACTTCAGCTCGGGCCGTATTCAACTCACGG
mfsA-cDNA-LYT10   CTCCTGGCGATTACGCAATATTCGATCCTGACTTCAGCTCGGGCCGTATTCAACTCACGG
mfsA-cDNA-NIH2624 CTCCTGGCAATCACGCAATATTCGATCCTGACTTCAGCTCGTGCCATATTCAACTCACGA
                  ******** ** ***************************** *** ************* 
                  1021                                                      1080
mfsA-DNA-LYT10    TTTCATTTAACGACTGCCCTAGTATCGGGTCTCTTCTACCTCGCTCCAGGTGCCGGGTTC
mfsA-cDNA-LYT10   TTTCATTTAACGACTGCCCTAGTATCGGGTCTCTTCTACCTCGCTCCAGGTGCCGGGTTC
mfsA-cDNA-NIH2624 TTTCATTTAACGACTGCCCTAGTATCGGGTCTCTTCTACCTCGCTCCAGGTGCCGGGTTC
                  ************************************************************
                  1081                                                      1140
mfsA-DNA-LYT10    CTGATAGGCAGTCTCGTCGGCGGTAAGCTTTCGGATCGCACCGTTCGGAGCTACATAGTA
mfsA-cDNA-LYT10   CTGATAGGCAGTCTCGTCGGCGGTAAGCTTTCGGATCGCACCGTTCGGAGCTACATAGTA
mfsA-cDNA-NIH2624 CTGATAGGCAGTCTCGTCGGCGGTAAACTTTCGGATCGCACCGTTCGGAGATACATAGTA
                  ************************** *********************** *********
                  1141                                                      1200
mfsA-DNA-LYT10    AAGCGCGGATTCCGTCTCCCTCAGGATCGACTCCACAGCGGGCTCATCACATTGTTTGCC
mfsA-cDNA-LYT10   AAGCGCGGATTCCGTCTCCCTCAGGATCGACTCCACAGCGGGCTCATCACATTGTTTGCC
mfsA-cDNA-NIH2624 AAGCGCGGATTCCGTCTCCCTCAGGATCGACTCCACAGCGGGCTCATCACATTGTTCGCC
                  ******************************************************** ***
                  1261                                                      1320
mfsA-DNA-LYT10    GTAGTGCCCATAATCGCGGCGTTTTTCGCGGGCTGGGGGCTCATGGGCAGTTTTAACTGC
mfsA-cDNA-LYT10   GTAGTGCCCATAATCGCGGCGTTTTTCGCGGGCTGGGGGCTCATGGGCAGTTTTAACTGC
mfsA-cDNA-NIH2624 GTAGTGCCCATAATCGCGGCGTTCTTCGCGGGCTGGGGGCTCATGGGCAGTTTTAACTGC
                  *********************** ************************************
                  1321                                                      1380
mfsA-DNA-LYT10    CTGAACACTTATGTGGCTGGTACGTTCCACACGCTCATTATTTATATTCCCTTTGTGTA
mfsA-cDNA-LYT10   CTGAACACTTATGTGGCTG-----------------------------------------
mfsA-cDNA-NIH2624 CTGAACACTTACGTGGCTGGTTTGTTCCACACCCTCATT--TATCTATTCCCTTTGTGTA
                  *********** *******
                  1381                                                      1440
mfsA-DNA-LYT10    CGTGCCCATAATAATGTTGTCTCTAACCGCGAGTAGAAGCCTTGCCACAGAACCGGTCTG
mfsA-cDNA-LYT10   ------------------------------------AAGCCTTGCCACAGAACCGGTCTG
mfsA-cDNA-NIH2624 CATGCCCACAATAA----------------------------------------------

                  1441                                                      1500
mfsA-DNA-LYT10    CAGTCATTGCAGGGAAGTATATGCTTCAATACTCCTTTTCTGCAGGAAGTAGTGCAGTCG
mfsA-CDNA-LYT10   CAGTCATTGCAGGGAAGTATATGCTTCAATACTCCTTTTCTGCAGGAAGTAGTGCAGTCG
mfsA-cDNA-NIH2624 ------------------------------------------------------------

                  1501                                                      1560
mfsA-DNA-LYT10    TTGATCTCGTGATAAACGCCCTCGGAGTTGGATGGACGTTCACGCTATGTATGGTACTTT
mfsA-cDNA-LYT10   TTGATCTCGTGATAAACGCCCTCGGAGTTGGATGGACGTTCACGCTATG-----------
mfsA-cDNA-NIH2624 ------------------------------------------------------------

                  1561                                                      1620
mfsA-DNA-LYT10    TCTTCCCACCTCTCAATGTACATGCTCACAGTTCTTACAGGTGTGGTTGCTTCGACTATA
mfsA-cDNA-LYT10   -----------------------------------------TGTGGTTGCTTCGACTATA
mfsA-cDNA-NIH2624 ------------------------------------------------------------

                  1621                                                      1680
mfsA-DNA-LYT10    GCTGGATTGATCACGGCGGCCATCGCAAGGTGGGGGATAAATATGCAAAGATGGGCAGAA
mfsA-cDNA-LYT10   GCTGGATTGATCACGGCGGCCATCGCAAGGTGGGGGATAAATATGCAAAGATGGGCAGAA
mfsA-cDNA-NIH2624 ------------------------------------------------------------

                  1681                      1707
mfsA-DNA-LYT10    AAAGGCTTTCAATATGCCTACCCAATAG
mfsA-cDNA-LYT10   AAAGGCTTTCAATATGCCTACCCAATAG
mfsA-cDNA-NIH2624 ----------------------------
```
