# Supplementary material for: Improving itaconic acid production through genetic engineering of an industrial Aspergillus terreus strain
Source: Microb Cell Fact. 2014 Aug 11;13:119. doi: 10.1186/s12934-014-0119-y (PMC4251695; doi:10.1186/s12934-014-0119-y)
Supplement: Additional file 9: Figure S8. — Sequence alignment of MFS from A. terreus NIH 2624 (up) and A. terreus LYT10 (down) (at the protein level). [file 12934_2014_119_MOESM9_ESM.pdf]

Additional file 9

Figure S8 Sequence alignment of MFS from *A. terreus* NIH2624 (up) and *A. terreus* LYT10 (down) (at the protein level)

```
              1                                                          60
MFS-NIH2624   MGHGDTESPNPTTTTEGSGQNEPEKKGRDIPLWRKCVITFVVSWMTLVVTFSSTCLLPAA
MFS-LYT10     MSHGDTESPNPVTSTEGSGQSEPEKRGPDIPLWRKCVITFVVSWMTLVVTFSSTCLLPAA
              *.*********.*:******.****:* ********************************

              61                                                        120
MFS-NIH2624   PEIANEFDMTVETINISNAGVLVAMGYSSLIWGPMNKLVGRRTSYNLAISMLCACSAGTA
MFS-LYT10     PEIAGEFDMTVETINISNAGVLIAMGYSSLIWGPMNKLIGRRTSYNLAISMLCACSAGTA
              ****.*****************:***************:*********************

              121                                                       180
MFS-NIH2624   AAINEEMFIAFRVLSGLTGTSFMVSGQTVLADIFEPVYRGTAVGFFMAGTLSGPAIGPCV
MFS-LYT10     AAINEEMFIAFRVLSGLTGTSFMVSGQTVLADIFEPVYRGTAVGFFMAGTLSGPAIGPCV
              ************************************************************

              181                                                       240
MFS-NIH2624   GGVIVTFTSWRVIFWLQLGMSGLGLVLSLLFFPKIEGNSEKVSTAFKPTTLVTIISKFSP
MFS-LYT10     GGIIVTFTSWRVIFWLQLAMSGLGLVLSLLFFPKVEALSEKASTASKPTTLVTIISKFSP
              **:***************.***************:*. ***.*** **************

              241                                                       300
MFS-NIH2624   TDVLKQWVYPNVFLADLCCGLLAITQYSILTSARAIFNSRFHLTTALVSGLFYLAPGAGF
MFS-LYT10     TDVLKQWVYPNIFLADLCCGLLAITQYSILTSARAVFNSRFHLTTALVSGLFYLAPGAGF
              ***********:***********************:************************

              301                                                       360
MFS-NIH2624   LIGSLVGGKLSDRTVRRYIVKRGFRLPQDRLHSGLITLFAVLPAGTLIYGWTLQEDKGDM
MFS-LYT10     LIGSLVGGKLSDRTVRSYIVKRGFRLPQDRLHSGLITLFAVLPAGTLIYGWTLQEGKGGM
              **************** **************************************.**.*

              361                                                       420
MFS-NIH2624   VVPIIAAFFAGWGLMGSFNCLNTYVAGLFHTLIYLFPLCTCPQ-----------------
MFS-LYT10     VVPIIAAFFAGWGLMGSFNCLNTYVAEALPQNRSAVIAGKYMLQYSFSAGSSAVVDLVIN
              ************************** .   . ..

              421                                          465
MFS-NIH2624   ---------------------------------------------
MFS-LYT10     ALGVGWTFTLCVVASTIAGLITAAIARWGINMQRWAEKAFNMPTQ
```
